# Relationship between nitrapyrin and varying nitrogen application rates with nitrous oxide emissions and nitrogen use efficiency in a maize field

**DOI:** 10.1038/s41598-022-23030-1

**Published:** 2022-11-01

**Authors:** Azam Borzouei, Hedayat Karimzadeh, Christoph Müller, Alberto Sanz-Cobena, Mohammad Zaman, Dong-Gill Kim, Weixin Ding

**Affiliations:** 1 Agriculture Research School, Nuclear Science and Technology Research Institute (NSTRI), P. O. Box: 31485-498, Karaj, Iran; 2 Justus Liebig University Giessen, Giessen, Germany; 3 University College Dublin, Belfield, Ireland; 4 ETSI Agrónomos, Technical University of Madrid, Ciudad Universitaria, 28040 Madrid, Spain; 5 Soil and Water Management and Crop Nutrition, Joint FAO, IAEA Division of Nuclear Techniques in Food and Agriculture, P.O. Box 100, 1400 Vienna, Austria; 6 Wondo Genet College of Forestry and Natural Resources, Hawassa University, PO Box 128, Shashemene, Ethiopia; 7 Institute of Soil Science, Chinese Academy of Sciences, Nanjing, 210008 China

**Keywords:** Plant ecology, Climate sciences, Climate change, Climate-change mitigation

## Abstract

Reducing nitrogen losses can be accomplished by mixing fertilizers with nitrification inhibitors (NI). In some agricultural systems, increasing soil N supply capacity by the use of NI could lead to improved N use efficiency (NUE) and increased crop yields. This study examined the effect of different N rates and NI in maize in the north of Iran. The maize was fertilized with urea at three levels (69, 115 and 161 kg N.ha^−1^) alone or with nitrapyrin as NI. Increasing the N application rate resulted in a considerable rise in growing-season N_2_O emissions. When nitrapyrin was used, N_2_O emissions were dramatically reduced. NI treatment reduced N_2_O emissions in the growth season by 88%, 88%, and 69% in 69, 115, and 161 kg of N.ha^−1^, respectively. NI treatment reduced yield-scaled N_2_O emissions; the lowest quantity of yield-scaled N_2_O was found in 69 N + NI (0.09 g N_2_O–N kg^−1^ N uptake). Additionally, grain yield increased by 19%, 31% and 18.4% after applying NI to 69 N, 115 N, and N69, N115 and N161. Results showed that 115 N + NI and N69 treatments showed the highest (65%) and lowest (29%) NUEs, respectively. Finally, our findings show that NI can reduce N_2_O emissions while increasing NUE and yield, but that the application method and rate of nitrapyrin application need to be improved in order to maximize its mitigation potential.

## Introduction

By 2050, the global population is predicted to reach a staggering ten billion people. As a result, agricultural output must rise by 56% to meet the world's food needs^1^. Cereals account for the largest percentage of crops in the food supply. The best yielding cereal crop, maize, is grown in more than 170 nations on an area of around 194 million hectares, with an annual production of 1147.6 million metric tons^2^. Additionally, maize is commonly used as a food or processed food in numerous countries around the world.

The supply of sufficient nitrogen for cereal crops, which have a nitrogen use efficiency (NUE) of 25 to 30 percent is the biggest challenge for sustainable agricultural production. Only around 100 million tons of N is produced by the Haber–Bosch process worldwide^3^, while it has been estimated that plants require approximately 150 to 200 million tons of mineral N^4^. In plant biology, N is the most important macronutrient. It is one of the essential nutrient required in the greatest quantity for maize production, which serves as the primary building block for amino acids, chlorophyll, adenosine triphosphate (ATP), and nucleic acids^5^. Nearly 9–11 kg of N are needed to produce one ton of crop biomass^6^. Increased N treatment in maize increases biomass, grain yield, leaf area, and shelling percentage^7,8^.

The global nitrogen cycle is of major concern because it contributes to regional and global environmental challenges. Due to the rising use of fertilizer in croplands, agricultural activities account for approximately 60% and 10% of worldwide anthropogenic N_2_O and NO sources, respectively^9^.

Nitrous oxide (N_2_O), the major non-CO_2_ greenhouse gas emitted from soils, is created through nitrification and denitrification^10,11^. Nitrogen fertilizer addition to maximize crop yields generally boosts N_2_O production^12^. The net emission of greenhouse gases from farming activities might possibly be minimized by adjusting crop management practices to improve soil organic carbon (SOC) content^13^ and decrease N_2_O emissions^14,15^. High N application can stimulate nitrification and/or denitrification processes and so boost N_2_O emissions from croplands. In general, there is a considerable increase of both N_2_O emissions accompanying with N application rates in croplands^16^ and row-crop cultivations^17,18^. Hoben et al., (2011)^19^ showed a nonlinear exponentially increasing N_2_O response to N application rates from a maize-soybean rotation, but N_2_O emissions were not significantly reduced with decreasing nitrogen fertilizer application in a winter wheat-summer maize rotation farmed by Yan et al.^20^. Although increasing N application mainly increases N_2_O emission, the intensity and amount of its increase depend on agricultural systems, environmental conditions, the amount of nitrogen consumed, and many other factors.

Ammonium (NH_4_^+^) can be delayed from being converted to nitrate (NO_3_^-^) by nitrification inhibitors, such as nitrapyrin^21^. Nitrification rate and NO3- concentration can be reduced by this inhibitor, which reduces N2O emissions directly^22^. Many field studies have shown that nitrification inhibitors can reduce N_2_O emissions from the application of chemical fertilizers, farm effluents, and manure under a wide range of cropping and soil regimes by more than half^22–24^.

It was the primary goal of this study to examine the effect of varying quantities of nitrogen fertilizer on N_2_O emission, yield-scaled N_2_, and NUE, as well as maize yield in presence or absence of NI.

## Results

### N_2_O fluxes

**Figure 1:**
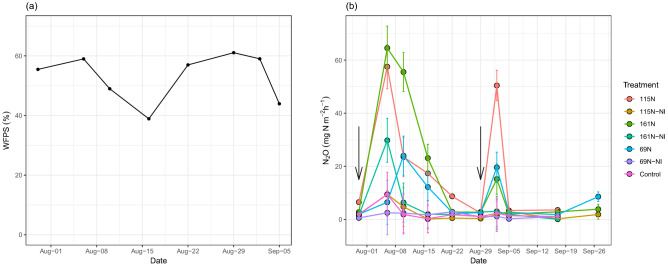
Temporal variations of soil water-filled pore space (WFPS) (**a**) and N_2_O emission (**b**) in the different levels of N application and with or without NI during the maize growth season in 2018. Vertical bars indicate the least significant difference (LSD) amount at p < 0.01, n = 3. Arrows denote time of N fertilization.

Fluxes of N_2_O emissions changed dynamics over the growth season. The results showed that N_2_O fluxes rose in direct correlation with the rate at which N was applied (Fig. 1b). Increasing the N application rate also increased the peak value of N_2_O emissions. N_2_O emissions peaked 60 days (seven days after N application; on August 6) and 87 days (four days after N application; on September 2) after sowing, respectively, during the growing period. When compared to treatments that do not include NI, the addition of NI could lower seasonal N_2_O emission fluxes. It also was observed that N_2_O emission peaks of N-NI were lower than that of N treatment.

### Cumulative N_2_O fluxes

**Figure 2:**
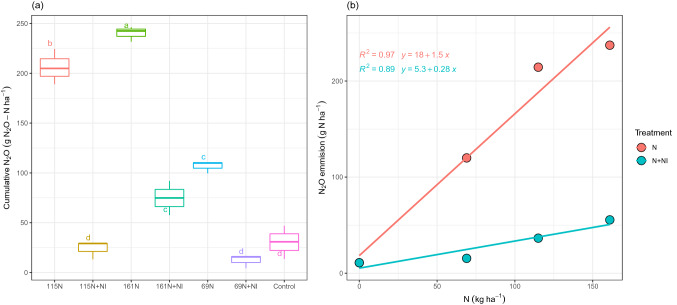
Cumulative N_2_O emission (**a**) and N_2_O emission versus N rates (**b**) at the different levels of N application and with or without NI during the maize growth season in 2018. Means with the same letters are not significantly different according to the least significant difference (LSD) at *p* < 0.01, n = 3.

Cumulative N_2_O was significantly affected by treatment (p ≤ 0.001); it fluctuated greatly among the treatments so that it varied from 12.2 to 240.1 g N_2_O–N ha ^−1^ in 69 N + NI and 161 N, respectively (Fig. 2a). So it was found that cumulative N_2_O emission is strongly affected by fertilizer N input; Great N application input resulted in more N_2_O emission. Compared to the not-application of NI, cumulative N_2_O emissions decreased by 88.6%, 88.4%, and 68.9% in the 69NI + NI, 115 N + NI, and 161 N + NI treatments respectively. As shown in Fig. 2b the relationship between N rate and N_2_O emission was positive and linear for both using NI (*p-value* < 0.05) and not-using NI (*p-value* < 0.05) conditions.

### Biomass, grain yield, N uptake and yield-scaled N_2_O emission

**Figure 3:**
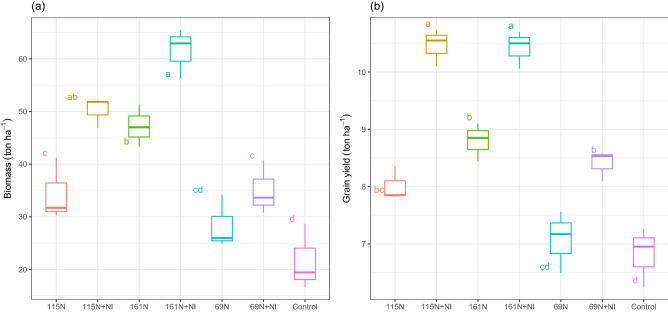
Biomass (**a**) and grain yield (**b**) of maize at the different levels of N application and with or without NI during the maize growth season in 2018. Means with the same letters are not significantly different according to the least significant difference (LSD) at *p* < 0.01, n = 3.

Maize biomass was significantly influenced by fertilizer treatment (p ≤ 0.001). The biomass showed an incremental trend due to increase of N rate. Results showed that in each level of N application, biomass increased by NI application (Fig. 3a). So compared to not-using NI, biomass increased by 23.6%, 45.9%, and 30.3% in 69 N + NI, 115 N + NI, and 161 N + NI treatments, respectively. Grain yield also was significantly affected by fertilizer treatment (*p* ≤ 0.001) and it increased in all treatments compared to the control. The highest grain yield increment was observed when using the 115 N + NI treatment so maize grain yield increased from 6.8 tons ha^-1^ (control) to 10.5 tons ha^-1^ (Fig. 3b). The grain yield increased significantly due to NI application; it increased by 18.8% in 69 N + NI, 30.6% in 115 N + NI, and 18.5% in 161 N + NI, compared to 69 N, 115 N, and 161 N, respectively.

**Figure 4:**
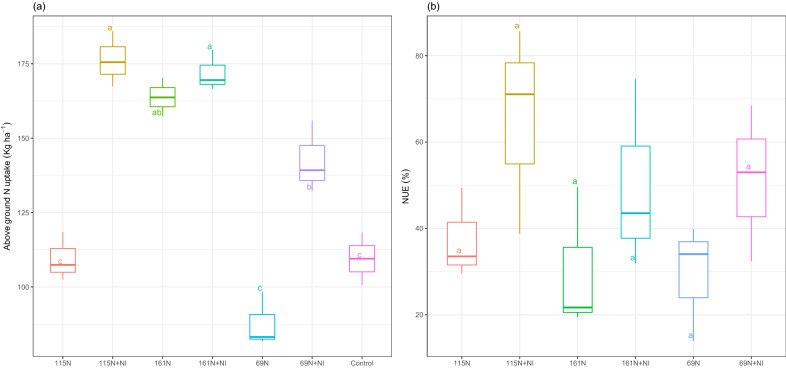
Above-ground N uptake (**a**) and nitrogen use efficiency (NUE) (**b**) of maize at the different levels of N application and with or without NI during the maize growth season in 2018. Means with the same letters are not significantly different according to the least significant difference (LSD) at *p* < 0.01, n = 3.

The fertilizer treatment had a significant impact on above-ground N uptake (*p* ≤ 0.001). Except 161 N + NI, above-ground N uptake increased significantly due to NI application (Fig. 4a). Results showed that above-ground N uptake increased by 62.3% in 69 N + NI, 61.1% in 115 N + NI, and 5% in 161 N + NI treatments than 69 N, 115 N, and 161 N respectively.

Nitrogen use efficiency (NUE) was not significantly affected by fertilizer treatment although in each N level, NI application increased NUE (Fig. 4b). Results indicated that NUE increased by 75.3% in 69 N + NI, 73.8% in 115 N + NI, and 65.5% in 161 N + NI compared to 69 N, 115 N, and 161 N treatments respectively. A positive and significant correlation was also observed among grain yield, above-ground N uptake, and NUE (Table 1).

**Table 1:** Key soil physiochemical properties at 0–30 cm at the beginning of the experiment.

Parameter	Unit	Value
pH		7.5
EC	(dS m^−1^)	1.1
Bulk density	(g cm^−3^)	1.27
Organic carbon	(%)	0.78
N	(%)	0.09
P	(g kg^−1^)	13.5
K	(g kg^−1^)	180
CEC	(meq/100 g)	17.1

*EC* Electrical conductivity, *N* Nitrogen, *P* Phosphorus, *K* Potassium, *CEC* Cation exchange capacity.

**Figure 5:**
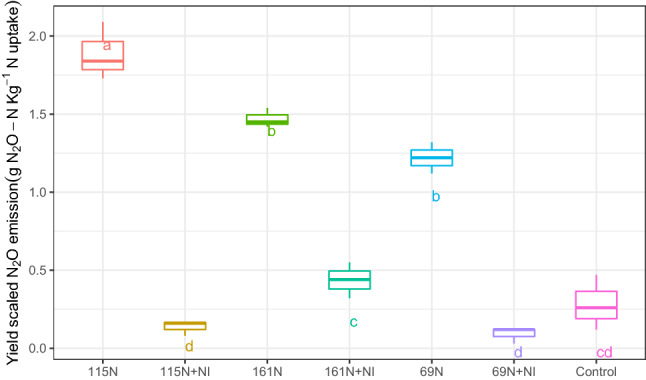
Yield-scaled N_2_O emission of maize at the different levels of N application and with or without NI during the maize growth season in 2018. Means with the same letters are not significantly different according to the least significant difference (LSD) at *p* < 0.01, n = 3.

Fertilizer treatment had a significant impact on yield-scaled N_2_O emission (p ≤ 0.001). The results indicated that in each level of N consumption, NI decreased yield-scaled N_2_O emission (Fig. 5) so that it decreased by 92.6%, 92.8%, and 70.3% in 69 N + NI, 115 N + NI, and 161 N + NI, respectively.

## Discussion

Nitrogen fertilization markedly influenced the soil N_2_O emission, although the effects of N fertilization were quite different in terms of nitrogen applications rates. In addition, it was shown that NI significantly reduced seasonal N_2_O emissions. N_2_O emissions are expected to rise if the N application rate is increased^25^. Nitrapyrin, a nitrification inhibitor, has been shown in several tests to be beneficial in reducing N_2_O losses^26,27^. If we compare these high mitigation efficiency results to those reported in the meta-analysis Gilsanz et al.^28^, it is clear that in these calcareous soils with low organic C content nitrification plays an important role in producing N_2_O. Soil NH_4_^+^ and nitrification/denitrification substrates could be the main cause of the changes in emission across fertilizer applications.

The nitrification and denitrification processes were shown to be most prevalent at WFPS values of 45–60% and 75–80%, respectively, according to Ding et al.^29^ The maximum WFPS (58 percent, Fig. 1a) and N_2_O emissions were detected in our study on August 6; additionally, the second peak of N_2_O emissions came on September 2 when WFPS was equal to 60 percent, indicating that N_2_O emissions may have mostly been caused by nitrification. N_2_O emission is influenced by soil temperature, WFPS, and mineral N content^30,31^. It has been reported that high soil temperatures (> 25 °C) facilitated N_2_0 emissions^32^. The reduction of NIs efficacy by increasing temperature is mainly due to microbial decomposition, stimulation of microbial activity, and volatilization of nitrapyrin^33^. As shown in Table 2, during the current study temperature exceed 25 °C so it can be concluded that the efficiency of NI was not maximum. However, according to the occurrence of high temperatures during the growth of corn in the study area in different years (1995–2020), it seems that the effect of temperature on the effectiveness of nitrapyrin in the study area in different years is constant and negligible. However, in cooler areas, the efficiency of Nitrapyren will probably be higher. Changes in mineral N concentration and WFPS were shown to be the primary causes of seasonal variations in N_2_O emissions during the maize growing season, according to our research. Guardia et al.^26^ discovered that irrigation management can play an important role in the effectiveness of NIs in lowering NO and N_2_O losses during the initial days following N fertilization, and their findings corroborated this. N_2_O emissions and WFPS have been found to have a strong association in other studies^32,34^.

**Table 2:** Temperature and precipitation data of study area for the growing season and long-time period (1995–2020).

Month	Temperature (°C)				Precipitation (mm)	
	Minimum		Maximum			
	Study period	Long-time	Study period	Long-time	Study period	Long-time
Jun	19.6	18.5	34.1	33.6	5	2.7
July	25.6	23.7	39.2	37.2	0	1.5
August	21.8	20.4	36.6	35.5	0	1.2
September	18.1	16.7	31.7	30.4	2.3	1.6

According to our findings, grain yield and biomass showed an increasing trend due to both N and NI applications. It was found that the larger aboveground biomass resulting from nitrapyrin consumption caused an increase in N crop uptake. On the other hand, nitrapyrin has the potential to dramatically enhance the uptake of N inorganics^35^. Nitrification inhibitor considerably increased the biomass yield of maize in other trials^36,37^. Application of a nitrification inhibitor enhanced grain production, which is consistent with previous studies by Ma et al.^38^ and Zhang et al.^39^. Zhang et al.^39^ found that nitrapyrin boosted vegetable yield by 13%, which they attributed to the compound's positive effects on plant growth and N uptake. However, there is some evidence to suggest that nitrification inhibitors can both raise soil NH_4_^+^ concentration and decrease soil NO_3_^-^ concentration, as well as boost crop yield, biomass, plant N absorption, and NUE. It is therefore possible to use nitrification inhibitors to increase yield and NUE in the wheat–maize cropping system while also reducing N_2_O emissions^32^.

In the current investigation, NI application resulted in a considerable increase in N uptake and NUE. In the prior study, an increase in N intake was also observed as a result of NI consumption^36^. Our results showed that NI treatment reduced yield-scaled N_2_O emissions in all N rates significantly. NI treatment has been shown to dramatically reduce yield-scaled N_2_O emissions by other researchers (for instance Ma et al.^38^; Zhang et al.^38,39^).

A yield-based analysis of N_2_O emissions can help estimate the environmental implications of intensive agriculture operations^40^. Emissions of N_2_O based on yield in this investigation ranged from 0.03 to 2.09 g N_2_O–N kg1. Under ideal conditions, Van Groenigen et al.^25^ found that N_2_O emissions were in the 5–15-g-N–N kg^−1^ range aboveground yield scaled. Li et al.^35^ found that nitrapyrin reduced yield-scaled N_2_O emissions by 42% during the trial period. Additionally, Dawar et al.^41^ found that urea treatment reduced yield-scaled N_2_O emissions by 47–52 percent, compared to urea treatment alone, when applied with nitrapyrin. It has been shown in the current study that yield-scaled N_2_O emissions have been reduced by roughly 85% (average of all N rates).

## Conclusion

The effectiveness of nitrapyrin in reducing yield-scaled emissions was often outperformed by N alone, according to our findings. The application of NI reduced N_2_O emissions by 69–89% at various levels of N. Due to NI application, the NUE was enhanced by 65.5–75.3%. In addition, NI was found to have a considerable impact on grain yield (18–31%) and biomass production (24–46%). Finally, our findings show that NI can reduce N_2_O emissions while increasing NUE and yield.

### Material and methods

A field experiment was conducted during 2018 growing season to evaluate the effect of applying different rate of urea with nitrapyrin (NI) at Nuclear Science and Technology Research Institute, Karaj, Iran. The average annual precipitation and evaporation in the study area are 247 and 2184 mm, respectively. The yearly average temperature is 14.4 °C, with a relative humidity of 53 percent. The climate is semi-arid with relatively cool winter and summer. The meteorological data during study period as well long-time (1995–2020) period is shown in Table 2. The experimental field had not been planted in the past few years. The soil texture was sandy clay loam (sand = 58.7%, silt = 20.10%, and clay = 21.20%) and the soil was classified as Typic Calcixerepts. The important soil parameters are reported in Table 3. Figure 1a also shows the seasonal changes in water-filled pore space (WFPS).

**Table 3:** Pearson correlation coefficients between Cumulative N_2_O, grain yield, biomass, Above-ground N uptake, Yield-scaled N_2_O emission and nitrogen use efficiency (NUE) in maize field at different rates of N and NI.

	Cumulative N_2_O	Grain yield	Biomass	Above-ground N uptake	Yield-scaled N_2_O emission	NUE
Cumulative N_2_O	1					
Grain yield	−0.11	1				
Biomass	0.1	0.84***	1			
Above-ground N uptake	−0.1	0.86***	0.82***	1		
Yield-scaled N_2_O emission	0.93***	−0.29	−0.12	−0.4	1	
NUE	−0.14	0.69***	0.55*	0.49*	−0.18	1

* and *** Significant at *P* < 0.05 and *P* < 0.001, respectively.

The experiment was carried out using a randomized complete block design (RCBD) with three replications. Fertilizer treatment included N69 (150 kg urea.ha^−1^; 69 kg N.ha^−1^), N115 (250 kg urea.ha^-1^; 115 kg N.ha^−1^), N161 (350 kg urea.ha^−1^; 161 kg N.ha^−1^), N69 + NI, N115 + NI, and N161 + NI. N0 was defined as a control plot that received zero N. The above-mentioned Ns were the total amount of nitrogen that was used in the experiment. Twenty-one plots were formed, each with a 14 m^2^ size (3.5 m × 4 m) and 1.5 m boundary. Each plot has five rows of plants spaced 0.7 m apart. On June 7, 2018, seeds were sowed at a distance of 10 cm and a density of 8 plants per square meter.

On July 30 and August 29, 2018, the N fertilizer was top-dressed so that half of the nitrogen fertilizer was used on each date. NI was added to the N fertilizer at a rate of 0.35% (W/W)^42,43^ and was surface applied by hand and then integrated into the cultivated layer using irrigation water. Because there was no rain during the growing season, 10-day intervals of surface irrigation were used.

### Labeled nitrogen experiment

The uptake of ^15^N-labeled fertilizer was monitored in small plots (1*4 m) within the main experiment area. Three replications of six treatments were used in a randomized full block design with micro plots. All micro plots received different rate of nitrogen with or without NI same as main plots, with the following rate treatment: 69 kg (^15^ N labeled) ha^−1^, 69 kg (^15^ N labeled).ha^−1^ + NI, 115 kg N.ha^−1^(^15^ N labeled), 115 kg (^15^ N labeled).ha^−1^ + NI, (v)161 kg (^15^ N labeled) ha^−1^ and 161 kg (^15^ N labeled) + NI. Urea 46 percent and urea ^15^N enriched (5 atom percent excess ^15^N) were used in the formulation of fertilizer solutions for all isotopic and non-isotopic treatments.

We used both ^15^N-labeled and non-labeled fertilizers, which were dissolved in water and applied to the specified area due to the small size of the isotopic subplots (1 × 4 m) and the ability to move ^15^N material out of them and ensure uniform distribution of fertilizers (by hand sprinkler). Non-isotopic plants were sprinkled with 15 L of water containing all of the pollutants. There were also ^15^N-labeled fertilizer and chemical mixtures in 3L of water, which were then distributed among isotopic plants in isotopic subplots for the experiment as a whole.

### Sampling and measurements of gas and soil

A closed-chamber approach was utilized to determine the fluxes of N_2_O in each plot, and an Agilent 7890B gas chromatograph with an electron capture detector was used to measure the concentrations of N_2_O. (ECD). 58 days were spent conducting gas samplings from July 30th to September 27th, 2018. For the gas collection device, we used an organic glass chamber with an embedded stainless steel base (0.4 m wide, 0.04 m long, and 0.04 m high). Between 8:00 a.m. and 12:00 a.m., each treatment plot was sampled 30 min. One sample was collected from each chamber and 3 samples were measured for each plot. After using an injectable syringe, N_2_O concentration was measured as quickly as feasible in the laboratory. Linear regression equations were used to obtain the average rate of change in gas concentration, which was then used to calculate the gas-fluxes using Eq. 1:

1 $$F = \rho \times \left( {V/A} \right) \times \left( {\Delta C/\Delta t} \right) \times \left[ {273/273 + T} \right]$$

F: N2O flux (g m^−2^ h^−1^).V: volume of the chamber (m^3^).Δc/Δt: average rate of concentration change over time (ppm v h^−1^); ρ:density of N_2_O (mg m^−3^).A: base area of the chamber (m^2^).T: chamber temperature (°C)

There was no filtering criterion used in static chamber measurements when the maximum concentration difference was less than the gas specific GC detection limit (i.e. 20 ppm for CO_2_, 20 ppb for CH_4_, and 0.80 ppb for N_2_O), in which case the R^2^ threshold for accepting N_2_O fluxes was set at 0.80 (p0.1)^44^. Ten percent of N_2_O fluxes were omitted from later data analysis in order to meet quality standards. In order to estimate the total amount of N_2_O emissions, we used the following equation:

2 $$\mathop \sum \limits_{i = 1}^{n} \left( {F_{i} + F_{i + 1} } \right)/2 \times \left( {t_{i + 1} - t_{i} } \right) \times 24$$

Fi, Fi + 1: the ith and (i + 1)th measured values of N_2_O flux (g N_2_O–N m^−2^ h^−1^); ti and ti + 1: days when the ith and (i + 1)th measurements of N_2_O flux were taken; n: total number of the measurements.

According to Van Groenigen et al.^25^, the yield-scaled N_2_O emission was computed based on aboveground N absorption and cumulative N_2_O emissions.

3 $$Yield\, -\, scaled{\mkern 1mu}\, N_{2} O{\mkern 1mu}\, emissions\,\left( {gN_{2} O - Nkg^{{ - 1}} \,above{\mkern 1mu} \,ground{\mkern 1mu}\, Nuptake} \right) = \frac{{Cumulative{\mkern 1mu}\, N_{2} O{\mkern 1mu}\, emissions}}{{above{\mkern 1mu}\, ground{\mkern 1mu}\, N{\mkern 1mu}\, uptake}}$$

### Determination of crop yield and ^15^N analysis

The ears and straw were sorted and weighed after harvesting. Drying ears for four days at 65 °C yielded grain yields. According to Lynch and Barbano^45^, the Kjeldahl digestion method was used to estimate plant nutrient uptake. The above ground N content was computed by adding the N mass measured in grain and straw from each plot together. Using an emission spectrometer, the total N and ^15^N/^14^N isotope ratio of dried plant tissues were measured. The IAEA's guidelines for ^15^N recovery in plants were followed when performing the calculations^46^. NUE was also calculated using Eq. (4)^47^.

4 $$NUE\left( \% \right) = \frac{{^{15} N\,atoms\,\%\,in\,plant}}{{^{15} N\,atoms\,\%\,in\,fertilizer}}$$

### Statistical data analysis

Analysis of data was carried out using the GLM procedure in the SAS 9.4 environment (SAS Institute Inc, Cary, NC, USA). The least significant difference (LSD) at p 0.01 was used for the mean comparison. The Shapiro–Wilk test was used to determine whether the data had a normal distribution. The ggplot2 package in R (4.1.2, Boston, Massachusetts, United States) was used to create all of the figures.

### Ethical approval

We confirm that all the experimental research and field studies on plants (either cultivated or wild), including the collection of plant material, complied with relevant institutional, national, and international guidelines and legislation. All of the material is owned by the authors and/or no permissions are required.

## Data Availability

All data analyzed during this study are included in this published article and its supplementary information files. The datasets analyzed during the current study available from the corresponding author on reasonable request.
