# Author Correction: Relationship between nitrapyrin and varying nitrogen application rates with nitrous oxide emissions and nitrogen use efficiency in a maize field

**DOI:** 10.1038/s41598-023-28793-9

**Published:** 2023-01-30

**Authors:** Azam Borzouei, Hedayat Karimzadeh, Christoph Müller, Alberto Sanz-Cobena, Mohammad Zaman, Dong-Gill Kim, Weixin Ding

**Affiliations:** 1 Agriculture Research School, Nuclear Science and Technology Research Institute (NSTRI), P. O. Box: 31485-498, Karaj, Iran; 2 Justus Liebig University Giessen, Giessen, Germany; 3 University College Dublin, Belfield, Ireland; 4 ETSI Agrónomos, Technical University of Madrid, Ciudad Universitaria, 28040 Madrid, Spain; 5 Soil and Water Management and Crop Nutrition, Joint FAO, IAEA Division of Nuclear Techniques in Food and Agriculture, P.O. Box 100, 1400 Vienna, Austria; 6 Wondo Genet College of Forestry and Natural Resources, Hawassa University, PO Box 128, Shashemene, Ethiopia; 7 Institute of Soil Science, Chinese Academy of Sciences, Nanjing, 210008 China

Correction to: *Scientific Reports* https://doi.org/10.1038/s41598-022-23030-1, published online 01 November 2022

The original version of this Article contained an error in Figure 2b, where “kg ha-1” was incorrectly given as “kg ha-2” in Figure 2b, unit of horizontal axis. The original Figure 2 and accompanying legend appear below.

The original Article has been corrected.

**Figure 2:**
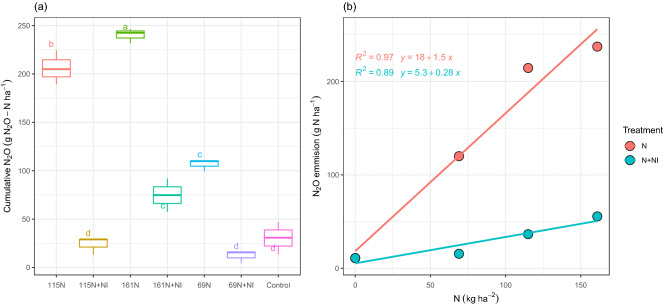
Cumulative N_2_O emission (**a**) and N_2_O emission versus N rates (**b**) at the different levels of N application and with or without NI during the maize growth season in 2018. Means with the same letters are not significantly different according to the least significant difference (LSD) at *p* < 0.01, n = 3.

